# Performance and usefulness of the Hexagon rapid diagnostic test in children with asymptomatic malaria living in the Mount Cameroon region

**DOI:** 10.1186/1475-2875-7-89

**Published:** 2008-05-22

**Authors:** Samuel Wanji, Helen K Kimbi, Joan E Eyong, Nicholas Tendongfor, Judith L Ndamukong

**Affiliations:** 1 Research Foundation for Tropical Diseases and the Environment (REFOTDE). P. O. Box 474, Buea, Cameroon; 2 Department of Biochemistry and Microbiology, Faculty of Science, University of Buea, P.O. Box 63, Buea, Cameroon; 3 Department of Plant and Animal Sciences, Faculty of Science, University of Buea, P. O. Box 63, Buea, Cameroon

## Abstract

**Background:**

Rapid and correct diagnosis of malaria is considered an important strategy in the control of the disease. However, it remains to be determined how well these tests can perform in those who harbour the parasite, but are asymptomatic, so that rapid diagnostic tests (RDTs) could be used in rapid mass surveillance in malaria control programmes.

**Methods:**

Microscopic and immunochromatographic diagnosis of malaria were performed on blood samples from the hyperendemic Mount Cameroon region. Thin and thick blood films were stained with Giemsa and examined under light microscopy for malaria parasites. The RDT was performed on the blood samples for the detection of *Plasmodium *species. In addition, the performance characteristics of the test were determined using microscopy as gold standard.

**Results:**

Results revealed 40.32% to be positive for microscopy and 34.41% to be positive for the RDT. Parasites were detected in a greater proportion of samples as the parasite density increase. *Plasmodium falciparum *was the predominant *Plasmodium *species detected in the study population either by microscopy or by the RDT. Overall, the test recorded a sensitivity and specificity of 85.33% and 95.05% respectively, and an accuracy of 91.40%. The sensitivity and specificity of the RDT increased as parasite densities increased.

**Conclusion:**

The Hexagon Malaria Combi™ test showed a high sensitivity and specificity in diagnosing malaria in asymptomatic subjects and so could be suitable for use in mass surveillance programmes for the management and control of malaria.

## Background

The cosmopolitan populations of some areas of the Mt. Cameroon region are often infected with *Plasmodium *species and the symptomless carrier status at any given time in children ranges from 60–100% [1]. This asymptomatic state forms an important source of continuous malaria transmission in the area. Although asymptomatic parasitaemia may be beneficial in inducing and sustaining partial immunity against malaria, a significant drawback is the suppression of haematocrit levels [2], thrombocytopaenia [3] and a negative effect on the cognitive function and school achievement of children [4,1]. The Mt. Cameroon region is hyperendemic for malaria with *Plasmodium falciparum *being the predominant *Plasmodium *species, although it co-exists with the non-falciparum species *Plasmodium malariae*, and *Plasmodium ovale*.

Generally in Cameroon, the best method of malaria control adopted by the National Malaria Control Programme is the early detection of parasites in symptomatic people followed by treatment with artemisinin combination therapies (ACTs) to curb the disease burden. Therefore, the malaria control programme in Cameroon usually leaves out individuals chronically infected with malaria parasites, but showing no symptoms.

The detection of malaria parasites by light microscopy of Giemsa-stained thick and thin blood films remains the standard laboratory method for the diagnosis of malaria [5] in many malaria endemic countries. However, there are problems and limitations associated with the reliance on the microscopic diagnosis of malaria. These include the fact that the technique is exacting normally requiring at least 60 minutes [6] from specimen collection to results and depends absolutely on good techniques (blood film preparation, preparation of stain), reagents, the state of the microscopes and most importantly the level of training of the microscopist [7]. Considering the fact that that malaria, especially that caused by *Plasmodium falciparum*, can lead to a rapid deterioration in the state of the patient, it is important to find ways of diagnosing the disease in the shortest possible time.

Rapid diagnostic tests (RDTs) have been used since the early 1990's for the detection of malaria parasites in humans. Several studies focusing on the detection of histidine rich protein-2 (HRP-2) and *Plasmodium *lactate dehydrogenase (pLDH) have been used for several years and have been well received with RDTs showing high sensitivities and specificities compared to microscopy or polymerase chain reaction (PCR) [8-18]. Nonetheless, there are limitations that affect the accuracy of test results obtained. Some RDTs detect only HRP-2 of *P. falciparum *and cannot detect mixed infections.

RDTS have been primarily evaluated for their performance in well-controlled research settings using sick people as subjects with either microscopy or PCR as gold standard. It remains to be determined how well these tests can perform in those who harbour the parasite but are not sick. Such that, these tests could be recommended for rapid mass surveillance in malaria control programmes so as to reduce the transmission and consequently burden of the disease. In order for any control programme to be effective in reducing disease prevalence, it should be both preventive and curative.

One of the RDTs that were recently introduced into the market is the Hexagon Malaria Combi™ and so far its performance characteristics and usefulness have not been evaluated in the Mt Cameroon Region. The main objective of this study was therefore to evaluate the performance characteristics and usefulness of Hexagon Malaria Combi™ test in detecting *P. falciparum *only, non-falciparum species and mixed infections with *P. falciparum *in a population that was primarily asymptomatic for malaria in the hyperendemic Mt Cameroon region of South West, Cameroon.

## Methods

### Study site

Samples were collected from school children at six different localities (Bonakanda, Likoko Membea, Meanja, Mutengene, Debundscha and Tiko) in the in the Mt Cameroon region. This region stretches from the Atlantic Ocean at the Golf of Guinea and culminates at an altitude of 4,100 m above sea level. The equatorial climate has been modified by the double influence of the ocean and mountain. Temperatures range from 18°C to 35°C. Rainfall ranges from 1,500 mm–11,100 mm. This region has a mean humidity of 88%. About 1.3 million people live around the Mount Cameroon region. The population is cosmopolitan, made up of indigenous Bakweri people and immigrants from other areas around the country and foreigners (mostly Nigerians) attracted by the rich volcanic soil for agricultural processes and other job opportunities. The terrain is fairly hilly with many streams. At lower altitudes the extensive plantations of bananas, palms, rubber and the creation of agglomeration have totally replaced the primary forest.

### Ethical consideration

Ethical clearances for the study were obtained from the South West Provincial Delegations of Public Health and Basic Education. The ethics committee of the Tropical Medicine Research Station, Kumba, South-West Province, Cameroon, approved the study. All pupils found to be infected were treated with artesunate/amodiaquine.

### Patients and sample collection

Sensitization visits were made by members of the research team to the localities to explain the objectives and advantages of the study to the teachers and the pupils. The study was carried out between March to June 2006. Children aged 4–16 years of both sexes in the six different localities were enrolled into the study. Informed consent was obtained from the parent/legal guardian of each child. The team of investigators moved to each of the six sites once every month for sample collection. Each child was observed for any clinical signs of malaria and his/her axillary was temperature recorded. Two mL of venous blood was collected from each consenting individual and used to prepare thick and thin blood films. The remaining blood was put into EDTA tubes, labeled accordingly, transported on ice blocks to the laboratory and stored at -20°C.

### Microscopy

The thick and thin blood films were stained with 10% Giemsa stain and read at ×100 (oil immersion objective) of the microscope. The *Plasmodium *species were detected with the help of bench aids for malaria diagnosis [19]. Slides were considered positive when asexual forms and/or gametocytes were found. For each positive slide, parasites were counted against 200 leucocytes and expressed as parasites/microlitre (μl) of blood, assuming a white blood cell (WBC) count of 8,000 leucocytes/μl of blood [5]. Parasite densities were classified into <500, 501–5,000 and >5,000 for low, moderate and high parasitaemia respectively.

Slides were considered negative if no parasites were seen after observing 100 high-powered fields. The microscopist was blinded to the result of the rapid test.

### Rapid immunochromatographic test

Briefly, the Hexagon Malaria Combi™ test is a rapid diagnostic test for the qualitative detection of pLDH (*Plasmodium *lactate dehydrogenase) released from *Plasmodium *species and is intended for the diagnosis of malaria. The test is designed to detect malaria caused by *P. falciparum *and the non-falciparum species (*P. malariae, P. ovale *and *P. vivax*). The expected species sensitivities are 90% for *P. falciparum*, 89% for *P. m vivax *while the specificity of the test is 99.5%.

The Hexagon Malaria Combi™ test kits and blood samples were kept on a bench to equilibrate to room temperature prior to use. The test with 20 μl of whole blood was performed in strict accordance with the manufacturers' instructions. Briefly, the required quantity of conjugate strips and washing strips were inserted into different rows of the microplate frame. One drop (30 μl) of diluent was dispensed into the conjugate wells and four drops into the washing wells. 20 μl of whole blood was added into each of the conjugate wells using disposable pipettes for each sample, mixed gently and allowed for one minute. Test strips were immersed into the conjugate wells and allowed for five minutes. Test strips were then transferred into the washing wells and allowed for 10–15 minutes for the clearance of haemoglobin. The results were then read using the manufacturers' guide. The standard reading time was 21 minutes.

### Data analysis

Once all the samples had been tested, sensitivity, specificity, accuracy and reliability of the Hexagon Malaria Combi™ test were estimated using microscopy as gold standard. The variables measured were the number of true positives (TP), number of true negatives (TN), number of false positives (FP), and the number of false negatives (FN).

Sensitivity was calculated as TP/(TP+FN), specificity was calculated as TN/(TN+FP), the positive predictive value (PPV) was calculated as TP/(TP+FP), and the negative predictive value (NPV) was calculated as TN/(FN+TN) [20]. Test accuracy, the proportion of all tests that gave a correct result, were defined as (TP +TN)/number of all tests. The overall measure of reliability was calculated as (TP × TN) - (FP × FN)/(TP + FN) (TN+ FP).

## Results

### Demographic characteristics of study population

A total of 186 school children were recruited for the study. Of this 98 (52.69%) were females while 88 (47.31%) were males. The majority (52.69%) of the children belonged to the age group 9–12 years while the least (21.51%) belonged to the 13–16 years age group (Table 1). The mean age of the study population was 9.86 years while the mean temperature was 36.53°C (Table 1). No cases of fever (temperature ≥ 37.5°C) or a history of fever within 48 hours before sample collection were found in the study population.

**Table 1:** Demographic characteristics of the study population.

Characteristics	Males			Females			Overall (%)
Age group (Years)	N° (%)	Mean age (Years)	Mean temp (°C)	N° (%)	Mean age (Years)	Mean temp (°C)	
4–8	22 (48.8)	6.4	36.7	26 (54.2)	6.8	36.5	48 (25.8)
9–12	48 (48.9)	10.6	36.5	50 (51)	10.4	36.5	98 (52.7)
13–16	18 (45)	13.31	36.5	22 (55)	13.5	36.5	40 (21.5)
Overall	88 (47.3)	10.1	36.6	98 (52.7)	10.2	36.5	186 (100)

Temp. = Temperature

### Prevalence of malaria in the study population by microscopy

Of the 186 blood samples collected, 75 (40.32%) were found to be positive for malaria by microscopy. Of these, 40 (53.33%) of these were positive for *P. falciparum*, 10 (13.33%) were positive for non-falciparum species while 25 (33.33%) were positive for a combination of both *P. falciparum *and non-falciparum species.

### Prevalence of malaria in the study population by microscopy

Using the rapid test, 64 (34.41%) of the 186 blood samples were positive for malaria. 35 (54.69%) of these were positive for *P. falciparum*, eight (12.50%) were positive for non- falciparum species and 21 (32.81%) were positive for *P. falciparum *and non-falciparum species (Table 2).

**Table 2:** Performance of Hexagon Malaria Combi™ rapid test relative to microscopy of different *Plasmodium *species at different parasite densities.

	*P. falciparum *only		Non-falciparum species		Mixed infections with *P. falciparum*	
Parasite density	Number positive by microscopy	Number positive by rapid test (%)	Number positive by microscopy	Number positive by Rapid test (%)	Number positive by microscopy	Number positive by rapid test (%)
<500	4	2 (50)	1	0 (0)	2	1 (50)
501–5,000	13	10 (76.9)	3	2 (66.7)	6	4 (66.7)
>5,000	23	23 (100)	6	6 (100)	17	16 (94.1)
Total	40	35 (87)	10	8 (80)	25	21 (84)

Generally, parasites were detected in a greater proportion of samples as the parasite density increased in the three categories of *P. falciparum*, non-falciparum species and mixed infections both microscopically and by using the rapid test. *Plasmodium falciparum *was the most predominant *Plasmodium *species detected either microscopically (86.67%) or using the rapid test (87.50%) in the study population (Table 2).

### Performance characteristics of Hexagon Malaria combi™ rapid diagnostic test

Overall, when compared with microscopy as the gold standard, 64 out of the 75 samples positive by microscopy were positive by the rapid test giving a sensitivity of 85.33%. 106 samples were negative for the test out of 111 negative by microscopy giving a specificity of 95.50%. The percentage of all true positive and all true negative rapid test result gave an accuracy of 91.40%. The reliability of the rapid test expressed as j-index was 0.81 (Table 3).

**Table 3:** Comparison of Hexagon Malaria Combi™ rapid test with reference microscopy for the detection of *Plasmodium *species

Result of rapid test	Result of microscopy			Sensitivity 95%CI	Specificity 95%CI	Accuracy (%)	Reliability
					
	Positive	Negative	Total				
Positive	64	5	69				
Negative	11	106	116	85.3 (80.3–90.4)	95.5 (92.5–98.5)	91.4	0.8
Total	75	111	186				

Generally, the sensitivity and specificity of the rapid test increased as parasite density increased for each of the three categories. At parasite densities of >5000, the rapid test recorded the highest sensitivities and specificities while performance characteristics dropped as parasite densities decreased. The rapid test was more sensitive (87.50%) and specific (98.00%) for detecting *P. falciparum *only than cases of non-falciparum and mixed infections (Table 4).

**Table 4:** Performance characteristics of Hexagon Malaria Combi™ rapid test at different parasite densities in detecting the different *Plasmodium *species.

Parasite density	Sensitivity % (95%CI)	Specificity % (95%CI)	Accuracy %	Reliability
*P. falciparum *only				
<500	50 (33.4–66.6)	83 (52–98)	66.6	0.6
501–5,000	76.9 (62.9–90.9)	87 (60–98)	97.5	0.8
>5,000	100 (99.7–100)	92 (82–97)	98	0.8
Total	87.50 (76.5–98.5)	98 (93–100)	95.6	0.8
Non-falciparum species				
<500	0 (0–94.5)	67 (43–85)	98	0.7
501–5,000	66.7 (34–99.3)	88(47–100)	93	0.7
>5,000	100 (99.7–100)	98.(89–100)	96.5	0.7
Total	80 (52.3–99.6)	92 (83–97)	94	0.8
Mixed infections with *Plasmodium falciparum (Plasmodium vivax *not included)				
<500	50 (28.6–71.4)	62 (46–75)	95.5	0.7
501–5,000	66.7 (46.51–86.8)	66(53–78)	92	0.7
>5,000	94.1 (46.51–99.9)	92 (82–97)	93.1	0.8
Total	84 (68.3–99.7)	95 (74–100)	89.5	0.8

## Discussion

The recent development of rapid and accurate tests for the detection of malaria is highly commendable. One of the major goals of developing such rapid tests was that these rapid tests should be handled with ease and accurately by relatively unskilled staff for prompt diagnosis of malaria especially in areas where microscopy may not be available or available and pose problems because of its limitations [5,6] especially in some parts of the endemic areas where there is no electricity.

The strength of the present study is that it was performed in a population that was primarily asymptomatic in the hyperendemic Mt Cameroon region using microscopy as gold standard. Although asymptomatic malaria may be beneficial in inducing and sustaining partial immunity against malaria, it may give rise to severe disease complicated by coma, acidosis or severe anaemia [21]. Thus to better manage the disease, it is worthwhile to screen both sick and 'healthy' people and treat them promptly. From a malaria transmission perspective in the Mt Cameroon region, RDTs can play a key role in better management of the disease.

Currently, the management of malaria by the National Malaria Control Programme in Cameroon is based on the detection of parasites in sick people followed by treatment with ACTs. This still places the population at risk because the majority of individuals infected without symptoms serve as a source of continuous malaria transmission in an area. In order for this control programme to be successful in curbing the disease burden, there should be no time waiting for people to fall sick before treatment, rather mass surveillance using rapid diagnostic tests to identify non-sick people and to map out areas which are potentially at risk would better reduce the burden of the disease.

This study compared the diagnosis of malaria parasites by Hexagon Malaria Combi™ rapid test and traditional microscopy and found that the two methods yielded comparable results. A total of 186 asymptomatic children were tested; blood films identified 75 (40.32%) of these as positives while the rapid test identified 64 (34.41%). The fact that some malaria infections detected by blood films were not rapid test positive may be explained by the fact that Hexagon Malaria Combi™ rapid test detects parasite lactase dehydrogenase (pLDH), produced by life parasites only. Probably some of these children might have taken chemo-prophylaxis at home for malaria prevention. The possible explanations for discrepancies in test results obtained by microscopy and rapid test includes (i) insufficient detection of low parasitaemia by the rapid test (ii) Hexagon Malaria Combi™ rapid test detects only live parasites producing pLDH (iii) sequestration of parasites and (iv) false-positive reactions. Thus the five samples falsely positive by the rapid test could have been due to reasons (ii) and (iv) above while the 11 samples falsely negative for the rapid test could have been due to reason (i) above and probably the samples contained pLDH below the detection limit (20 ng/mL pLDH) of the rapid test.

When compared to other commercially available rapid tests (such as ParaSight F, ICT which detects only HRP-2), the Hexagon Malaria Combi™ rapid test is highly recommendable because it can detect all the species of *Plasmodium *(*P. vivax *not found in this area) present in the Mt. Cameroon region with good sensitivities and specificities at different parasite densities, thereby giving this tool a greater potential for use as an epidemiological tool for the control of malaria. Also, this test performed better than the OptiMAL™ rapid malaria test, which failed to detect asymptomatic malaria in an area of Thailand endemic for *P. falciparum *and *P. vivax *[22]. However, a major limitation of this rapid test was to differentiate between the non-falciparum species and to quantify the parasite density.

Unlike microscopy, which requires substantial training and experience, the Hexagon Malaria Combi™ rapid rest was performed well with minimal training. In addition, the rapid test can be performed in areas where electricity or microscopes are not available and the results can be obtained rapidly.

There are compelling reasons to justify the implementation of rapid tests for malaria diagnosis for asymptomatic carriers. First of all, microscopy when used for active malaria surveillance may not be accurate as active surveillance itself is a highly labour intensive activity that may provide minimal returns [23]. Secondly, waiting for people to fall sick before treatment is not very beneficial as malaria especially that caused by *Plasmodium falciparum *kills within a short period. Adults or the parents/legal guardians of children may be ignorant of the symptoms of malaria and may associate these symptoms to witchcraft or other superstitious beliefs.

However, savings in technician time, including time to prepare stained blood smears and microscopically examination of the slides, the lack of the need to purchase and maintain expensive equipment, and the ability to work in remote areas where electricity and other infrastructure is lacking, make the use of antigen detection test methods tremendously appealing.

The test has considerable potential for use in the management of malaria, provided it is made affordable. The cost of the test will clearly influence the extent to which it is deployed in any setting. To ensure that this useful tool can be made available to those in greatest need, the cost of the rapid test should not exceed U.S.$0.40/sample [6]. Unfortunately, unless such pLDH-based tests are produced in massive numbers, this price is unattainable and not practical for manufacturers to meet [24]. The current costs of the dipstick tests depend upon the location of the buyer and the volume ordered.

## Conclusion

In conclusion, the results obtained from this study demonstrate that the Hexagon Malaria Combi™ rapid test is suitable for use in mass surveillance programmes for the management and control of malaria in Cameroon, particularly in areas where the different species of *Plasmodium *co-exist. The combination of its high sensitivity and specificity for *P. falciparum*, non-falciparum species and mixed infections at the different parasite densities suggests that the majority of malaria cases will be detected using this rapid test.

## List of abbreviations

HRP-2: histidine rich protein 2; PCR: polymerase chain reaction; RDT: rapid diagnostic test; FN: false negative; TN: rue negative; FP: false positive; TP: true positive.

## Authors' contributions

EEJE: Data collection, analysis, interpretation and, preparation of manuscript. JLN: Data collection and reading of manuscript for important intellectual content. NT: Contributed in data analysis and reading manuscript for important intellectual content. HKK: Coordination and critically reading of manuscript for important intellectual content. SW: Conception of study and experimental design, provision of finances for the study, coordination, interpretation of data and critically reading the manuscript for intellectual content. All authors read and approved the manuscript.
